# Pupil Tracking for Real-Time Motion Corrected Anterior Segment Optical Coherence Tomography

**DOI:** 10.1371/journal.pone.0162015

**Published:** 2016-08-30

**Authors:** Oscar M. Carrasco-Zevallos, Derek Nankivil, Christian Viehland, Brenton Keller, Joseph A. Izatt

**Affiliations:** 1 Department of Biomedical Engineering, Duke University, Durham, North Carolina, United States of America; 2 Department of Ophthalmology, Duke University Medical Center, Durham, North Carolina, United States of America; Simon Fraser University, CANADA

## Abstract

Volumetric acquisition with anterior segment optical coherence tomography (ASOCT) is necessary to obtain accurate representations of the tissue structure and to account for asymmetries of the anterior eye anatomy. Additionally, recent interest in imaging of anterior segment vasculature and aqueous humor flow resulted in application of OCT angiography techniques to generate *en face* and 3D micro-vasculature maps of the anterior segment. Unfortunately, ASOCT structural and vasculature imaging systems do not capture volumes instantaneously and are subject to motion artifacts due to involuntary eye motion that may hinder their accuracy and repeatability. Several groups have demonstrated real-time tracking for motion-compensated *in vivo* OCT retinal imaging, but these techniques are not applicable in the anterior segment. In this work, we demonstrate a simple and low-cost pupil tracking system integrated into a custom swept-source OCT system for real-time motion-compensated anterior segment volumetric imaging. Pupil oculography hardware coaxial with the swept-source OCT system enabled fast detection and tracking of the pupil centroid. The pupil tracking ASOCT system with a field of view of 15 x 15 mm achieved diffraction-limited imaging over a lateral tracking range of +/- 2.5 mm and was able to correct eye motion at up to 22 Hz. Pupil tracking ASOCT offers a novel real-time motion compensation approach that may facilitate accurate and reproducible anterior segment imaging.

## Introduction

Optical coherence tomography (OCT) enables micron-scale, volumetric ocular imaging and has seen widespread clinical adoption [1,2]. In anterior segment OCT (ASOCT) imaging, volumetric acquisitions are necessary to obtain accurate representations of the tissue structure and morphology and to account for asymmetries of the anterior eye anatomy (e.g. irregular corneal thickness/curvature [3,4]). OCT angiography techniques have also been applied in the anterior segment to generate *en face* and 3D micro-vasculature maps of the human cornea-scleral limbus [5,6]. Unfortunately, OCT structural and vasculature imaging systems do not capture volumes instantaneously and are therefore subject to patient motion artifacts that may hinder their accuracy and repeatability.

While a subject’s voluntary eye motion may be mitigated with a fixation target, involuntary motion such as micro-saccades, drifts, or tremors [7,8] may still corrupt OCT volumetric data and associated *en face* summed volume projections (SVPs). In addition, the dynamics of human accommodation involve peak velocities in excess of 12 diopters/sec [9,10] and the accommodative state of the eye is variable even during steady fixation [11]. Experimental OCT systems with line rates in the MHz range [12,13] can significantly reduce motion artifacts, but associated increases in complexity and cost may limit their clinical applications. We and others have previously demonstrated methods for post-processing OCT volumetric data motion correction in the anterior segment [14] and retina [15–17]. However, these techniques required restrictive OCT acquisition schemes, including sequential orthogonal volume scans (X-fast, Y-fast scanning) or under-sampled radial B-scans, and complicated data reconstruction algorithms that have not yet been implemented in real-time. Moreover, large and rapid eye motion may result in discontinuities in OCT data which cannot be corrected in post-processing.

Real-time OCT tracking is an alternative technique that employs an auxiliary system to estimate and compensate for eye motion during OCT acquisition without the need for post-processing. Several groups have demonstrated retinal OCT tracking with a sensing beam [18,19] or scanning laser ophthalmoscope (SLO) [20] which allowed acquisition of densely sampled volumetric OCT data and phase-resolved angiograms [21] with greatly reduced motion artifacts. Unfortunately, these techniques may be difficult to extend to anterior segment OCT, in which the need to image over larger fields of view (compared to retinal imaging) with high A-scan sampling density results in slow frame rates and increased sensitivity to eye motion. Real-time presentation of motion corrected data to the physician would expedite clinical interpretation and could be crucial in applications in which the OCT data impacts decision-making in real-time, such as surgical guidance [22,23].

Pupil tracking is a well-established low-cost technique used to estimate a subject’s gaze and eye motion [24–28]. Compared to previous eye tracking implementations described above, pupil tracking relies on direct imaging of the subject’s pupil for eye motion estimation instead of imaging/sensing of the retina, thus making it an attractive option for real-time motion correction in anterior segment imaging. The image processing required to extract eye motion also does not necessitate graphics processing units (GPUs) for real-time operation, unlike computationally intensive motion estimation techniques in SLO-based retinal tracking [29]. Lastly, pupil/iris cameras are already commonplace in clinical-grade OCT systems, which would further facilitate the integration of pupil tracking for motion corrected ASOCT imaging.

In this report, we expand upon our previous work on pupil tracking for automated control of the beam entry position in retinal OCT [30] and demonstrate a pupil tracking system for real-time motion compensated swept-source ASOCT imaging. Pupil oculography hardware coaxial with the swept-source OCT enabled fast detection and tracking of the pupil centroid. The pupil tracking ASOCT system with a field of view of 15 x 15 mm achieved diffraction-limited imaging over a lateral tracking range of +/- 2.5 mm and was able to correct eye motion at up to 22 Hz. Pupil tracking ASOCT offers a novel real-time motion compensation approach that may facilitate anterior segment volumetric imaging.

## Materials and Methods

### OCT system design

**Fig 1:**
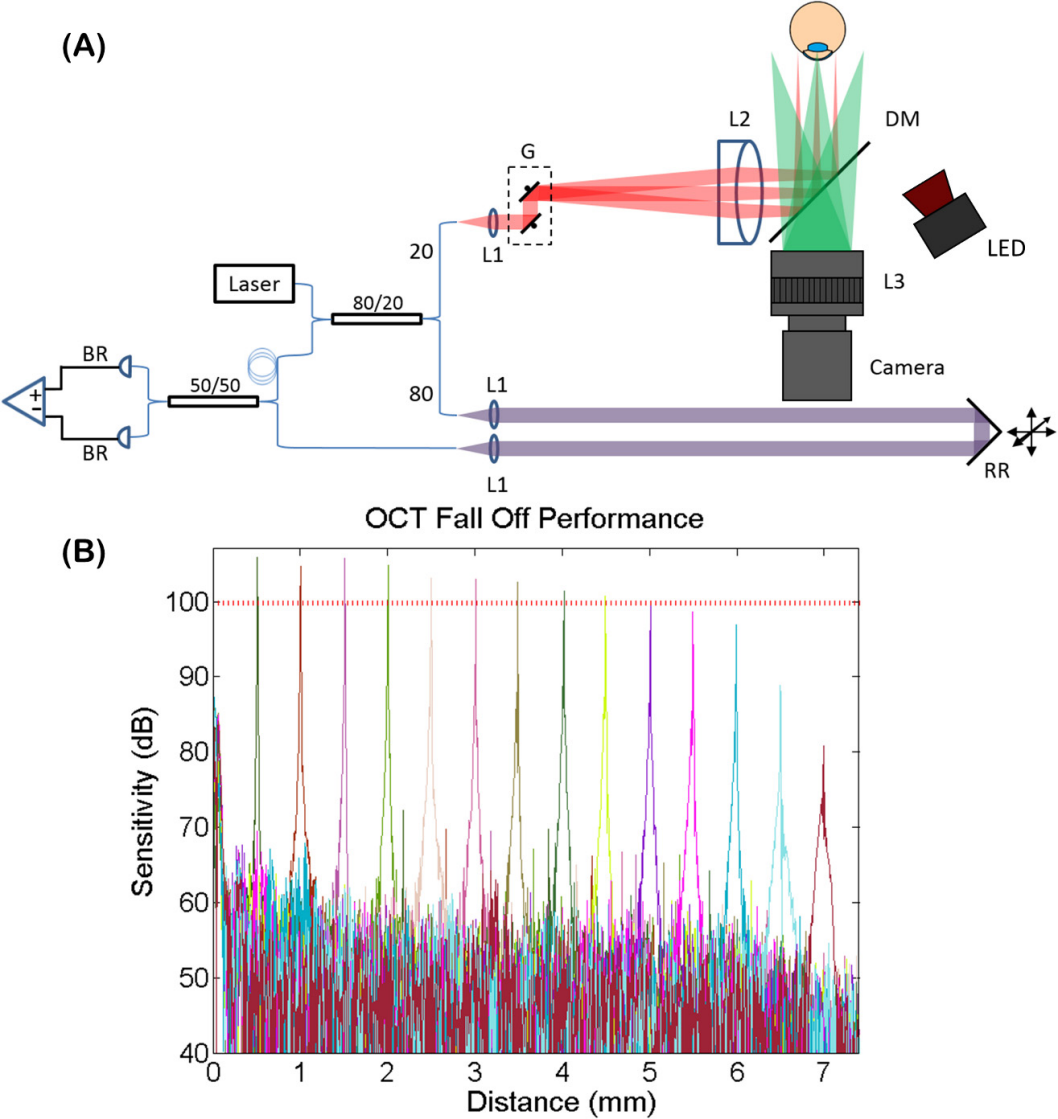
Optical coherence tomography system design and performance. (A) Diagram of swept-source anterior segment OCT system. G: galvanometer scanning mirrors, L: lens, RR: retro-reflector, DM: dichroic mirror, BR: balanced receiver. (B) OCT sensitivity fall off performance. Fall off of -6dB was measured at 4.6 mm. The red dashed line denotes -6 dB.

The custom swept-source ASOCT system depicted in Fig 1(A) used a 100 kHz swept-frequency laser (Axsun Technologies; Billerca, MA), centered at 1060 nm with a bandwidth of 100 nm, to illuminate a transmissive topology interferometer. The interferometric signal was detected with a balanced photoreceiver (Thorlabs, Inc.; Newton, NJ) and digitized at 800 MS/s (Alazar Tech Inc.; Pointe-Claire, QC, Canada). The sample arm included a collimator lens, galvanometer scanning mirrors (Cambridge Technology, Inc.; Bedford, MA), and a telecentric imaging lens with a working distance of 75 mm and a field of view (FOV) of 15 x 15 mm. The theoretical diffraction limited lateral resolution was 39.5 μm and the theoretical depth of focus was 6.7 mm. For pupil tracking, a hot mirror coupled a pupil camera and the OCT optical path (discussed in detail below). The measured sensitivity and -6 dB fall off of the OCT system (Fig 1(B)) was 104.5 dB and 4.6 mm, respectively. The optical clock provided by the source was digitized and interpolated to achieve an imaging range of 7.4 mm. Using a mirror as the sample, the axial resolution was measured at 8.3 μm. GPU-based software enabled real-time acquisition, processing, and rendering of volumetric data at a 100 kHz A-line rate.

### Pupil tracking system design

Video of the ocular pupil was acquired with a 1280 x 1024 monochromatic camera (Point Grey Research Inc.; Richmond, BC, Canada) with a pixel pitch of 4.8 μm. Real time images for tracking were acquired at a downsampled resolution of 320 x 256 pixels to achieve a frame rate of 500 Hz (limited by camera control software). The pixel size was 42 μm after magnification with a 16 mm focal length camera lens and the resulting camera FOV was 13.4 x 10.8 mm. An 850 nm IR light emitting diode (Thorlabs, Inc.; Billerca, MA) and a 50 mm focal length to decrease illumination divergence was placed 15 cm from the subject’s eye to enhance sclera/pupil contrast and to facilitate initial pupil tracing. A hot mirror with a reflectivity cutoff of 960 nm coupled the IR illumination and the pupil camera with the OCT optical axis. Incident light on the pupil camera was bandpass filtered with an optical bandpass camera filter centered at 850nm to prevent extraneous ambient and/or OCT back-reflected light from altering the histograms of the acquired pupil images.

The custom pupil tracking algorithm was implemented in C++ and has been previously published [30]. The 850 nm LED illumination was reflected by the iris and transmitted by the ocular pupil, yielding camera images with a bimodal intensity distribution that could be exploited for fast pupil segmentation using intensity thresholding. This approach was similar to previously reported eye tracking demonstrations [25]. Due to the stability of the IR illumination and the rejection of extraneous light by the camera bandpass filter, the intensity histogram of the acquired images did not vary substantially within each imaging session and the threshold value was determined once at the start of the session. Morphological closing with a 7x7 pixel kernel was applied to the binary image to remove extraneous dark regions (e.g. due to eye lashes, shadows) that could alter the pupil contour [28]. Connected component analysis, which has been previously applied in eye tracking applications [26], was used to identify the largest binary blob corresponding to the pupil. The pupil centroid location was estimated by calculating the center of mass of the detected binary blob. In real-time, the software displayed the location of the detected pupil centroid on processed displayed video frames. This segmentation approach was fast and did not necessitate GPU-accelerated software for real-time operation [28,25]. The tracking accuracy and repeatability of our pupil tracking algorithm were measured in our previous publication [30] and were 25 μm and 35 μm, respectively.

**Fig 2:**
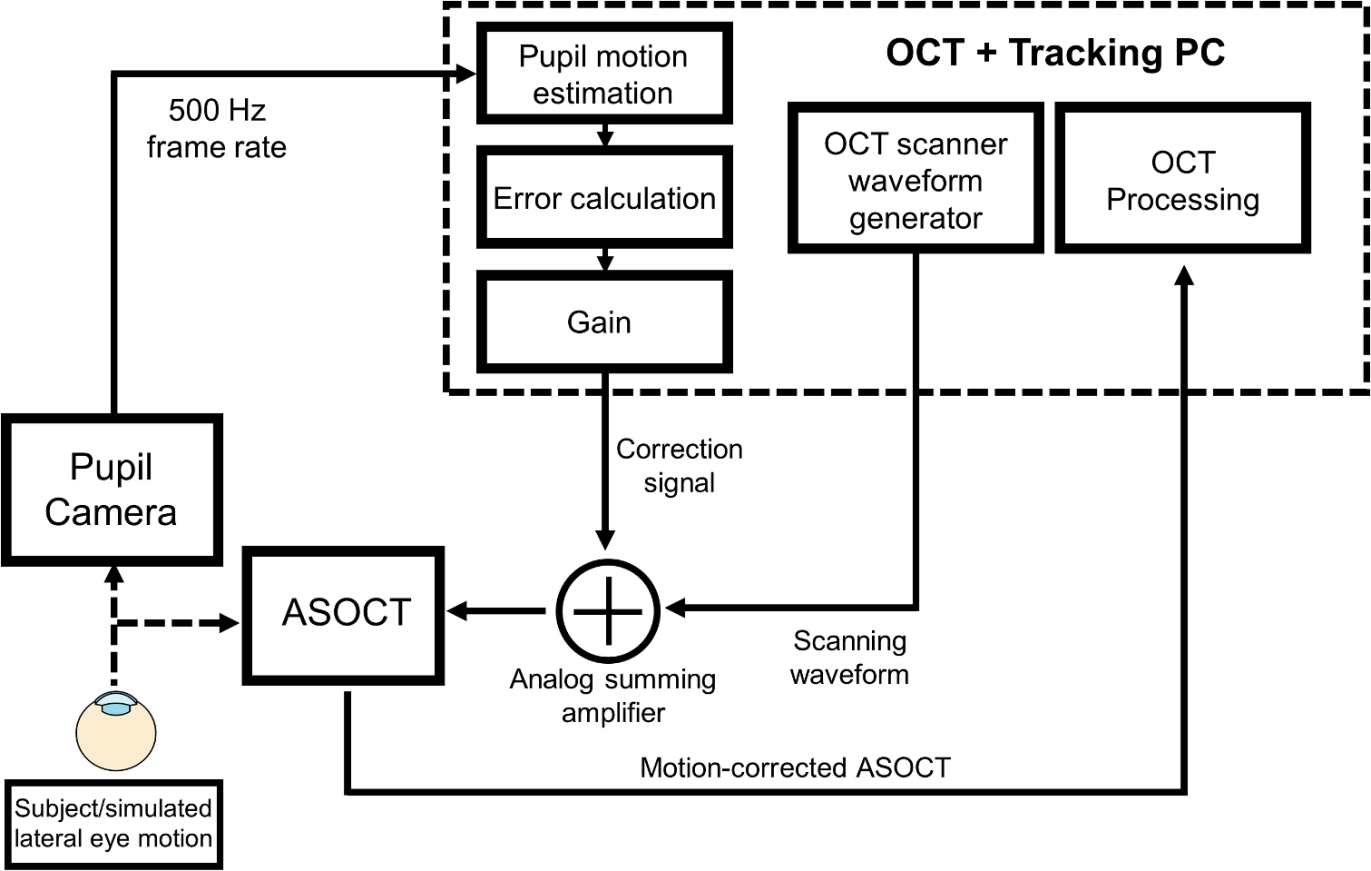
Schematic control system for pupil tracking ASOCT. The pupil camera imaged the ocular pupil at 500 frames/second and the acquired frames were used to estimate the eye’s lateral motion. The tracking algorithm calculated the deviation of the segmented ocular pupil from a designated reference position to estimate eye motion. Correction signals that were proportional to the magnitude of the estimated motion were then generated and summed with the OCT scanning waveforms using an analog summing amplifier.

Our pupil tracking implementation employed a proportional controller system to generate correction signals for the OCT scanners (Fig 2). The tracking algorithm computed the deviation of the calculated pupil centroid position from a designated central position (measured in camera pixels), multiplied this error signal by an appropriate gain, and generated voltage correction waveforms that were proportional to the magnitude of the measured error. The gain parameter *g* was determined using the following equation:

$$g=\frac{\Delta mm}{\Delta pix}\times \frac{\Delta \theta }{\Delta mm}\times \frac{volts}{\Delta \theta }\times \beta$$ (1)

where the first, second, and third ratios on the right hand side are the millimeter per pixel calibration for the pupil camera, the scanning mirror angle per millimeter calibration for the OCT telecentric scanner, and the voltage per angle calibration for the scanning mirrors, respectively. The parameter *β* was an adjustable gain factor used to minimize residual motion. The correction signals were summed with the X and Y channels of the scanning mirrors using an analog summing amplifier. The lateral tracking range was +/- 2.5 mm using a 15 x 15 mm FOV and was limited by the dichroic mirror.

**Fig 3:**
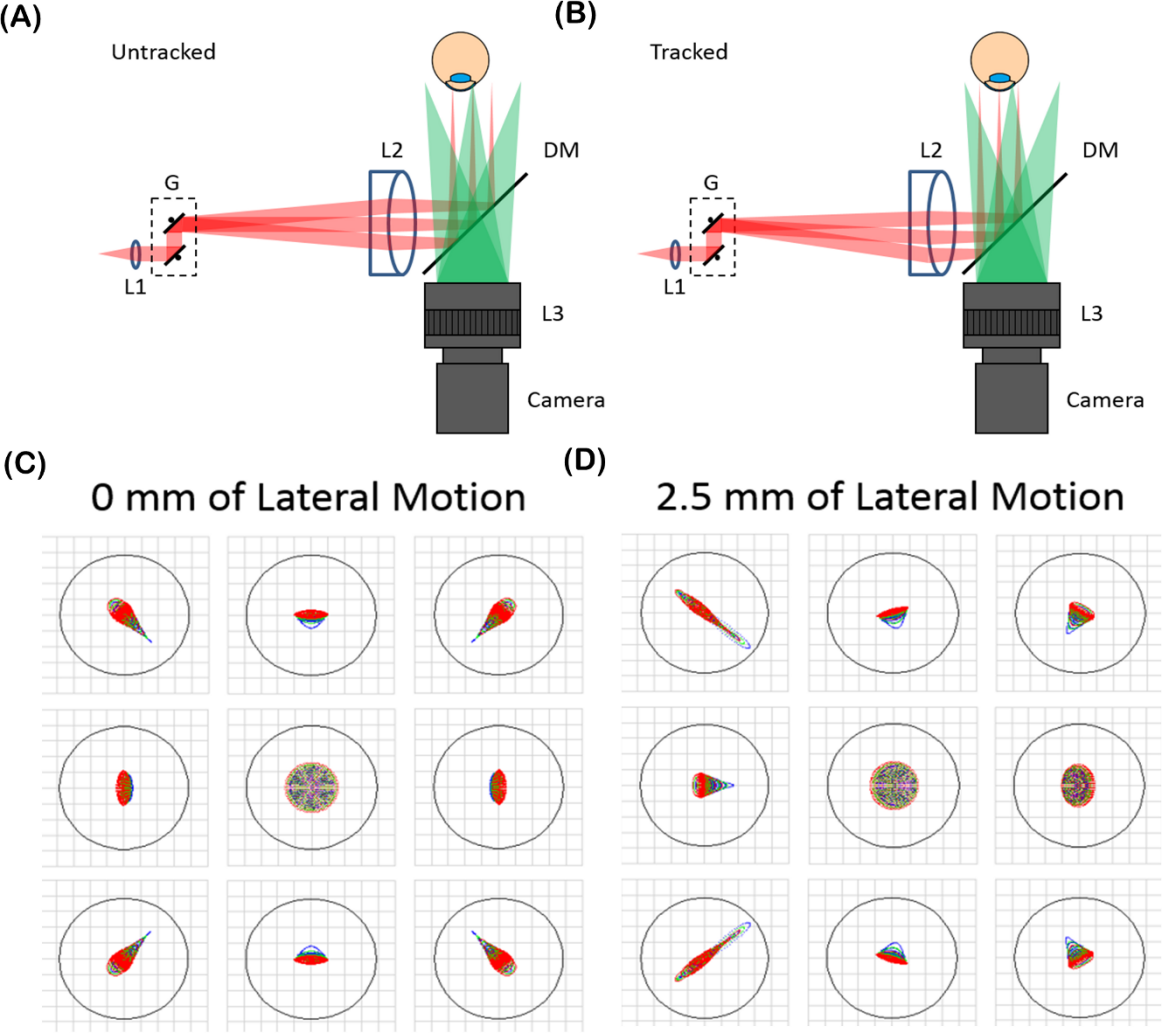
Optical performance of the anterior segment OCT system with and without tracking. (A-B) System diagram without tracking (A) and after 2.5 mm of lateral tracking (B). (C-D) Spot diagrams of the extrema of the +/- 15 mm tracking FOV. The airy radius was 39.5 μm as denoted by the black circles and the optical performance was diffraction limited.

The theoretical optical performance over the entire +/- 2.5 mm lateral tracking range was simulated in optical ray tracing software (Zemax, LLC; Kirkland, WA). Fig 3 shows the spot diagrams for the untracked and tracked configurations. The tracked scenario was simulated by offsetting the schematic eye [31] and OCT scan laterally by +/- 2.5 mm. Lateral resolution was diffraction limited (39.5 μm) throughout the entire tracking range while maintaining a constant OCT lateral FOV.

### Tracking system characterization setup

To quantitatively test the closed loop tracking performance, a 2D fast steering mirror (FSM) (Optics in Motion; Long Beach, CA) with a small angle bandwidth of 560 Hz was placed between the objective and a pupil phantom. The phantom was composed of a black circle on a white background (laser jet printed with 600 DPI resolution) to allow pupil detection using the tracking algorithm. The FSM was driven with an external waveform generator to simulate pupil phantom motion with arbitrary waveforms. The FSM driving waveform and the motion correction waveform generated by the tracking algorithm to compensate for the simulated motion were recorded using an oscilloscope with 1 ms resolution.

### Ethical considerations

Human subject imaging was conducted under a protocol approved by the Duke University Institutional Review Board. Prior to imaging, written consent from subjects was obtained after explanation of possible consequences and the nature of the study following the tenants of the Declaration of Helsinki. OCT optical power incident at the cornea was kept below 1.85 mW, which was below ANSI safety laser limit for 1060 nm. The IR illumination was 0.55 mW and was regarded as an extended source for ANSI power safety calculations. The combined optical power from both light sources never exceeded the maximum permissible exposure.

## Results

### Tracking system characterization

**Fig 4:**
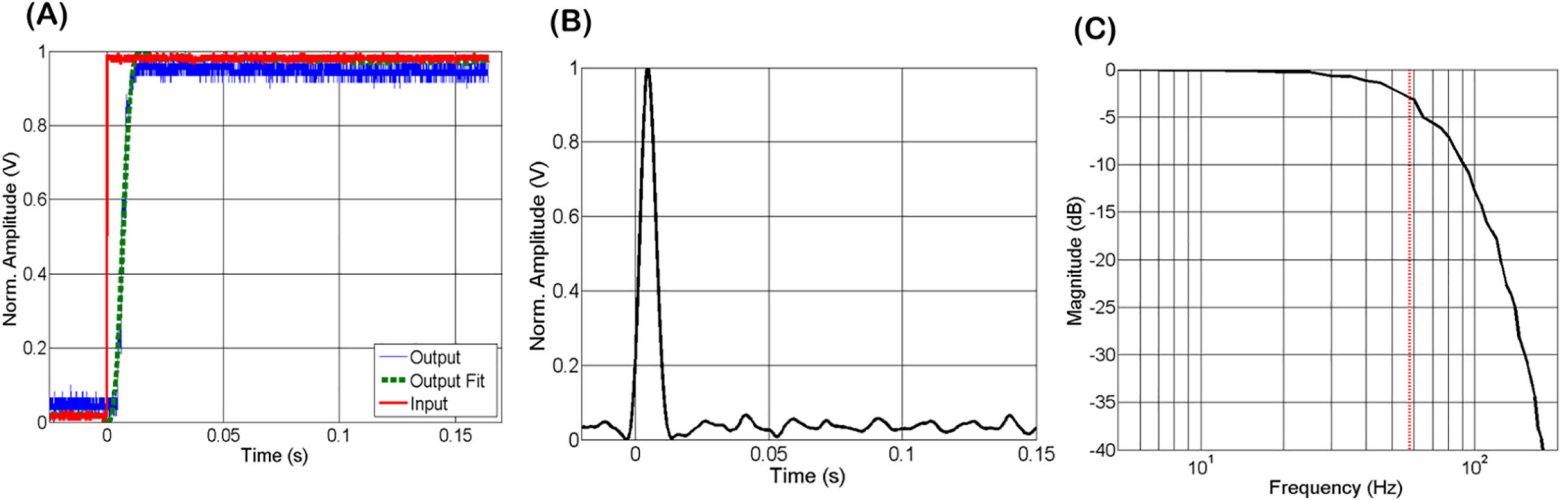
Characterization of pupil tracking system for a 3 mm step response. (A) Generated FSM driving waveform (input) and response waveform generated by tracking algorithm (output), and the fit to the output step function. (B) Impulse response after calculating the derivative of the step response. (C) Magnitude of the frequency response of the system estimated by calculating the Fourier transform of the impulse response. The -3 dB amplitude was measured at 58 Hz, as denoted by the dashed red line.

The impulse response of the tracking algorithm was analyzed using step waveforms to drive the FSM and simulate 1, 2 and 3 mm step motion lateral offsets of the phantom. The step response generated by the pupil tracking algorithm was measured before it was summed with the OCT scanning waveform. The derivative of the recorded step response was used to estimate the impulse response, and the magnitude of the Fourier transform of the impulse response was then calculated to estimate the system frequency response (Fig 4). The -3 dB amplitude roll-off of the transfer function was measured at 58 Hz for all three step inputs corresponding to 1, 2, and 3 mm of phantom lateral motion. Moreover, the control loop latency was measured by driving the FSM with a 1 Hz sinusoidal waveform to simulate sinusoidal motion of the pupil phantom. The cross-correlation of the FSM drive waveform and the response sinusoidal waveform generated by the tracking algorithm was measured and interpreted as the latency of the tracking algorithm, which was 4 ms.

**Fig 5:**
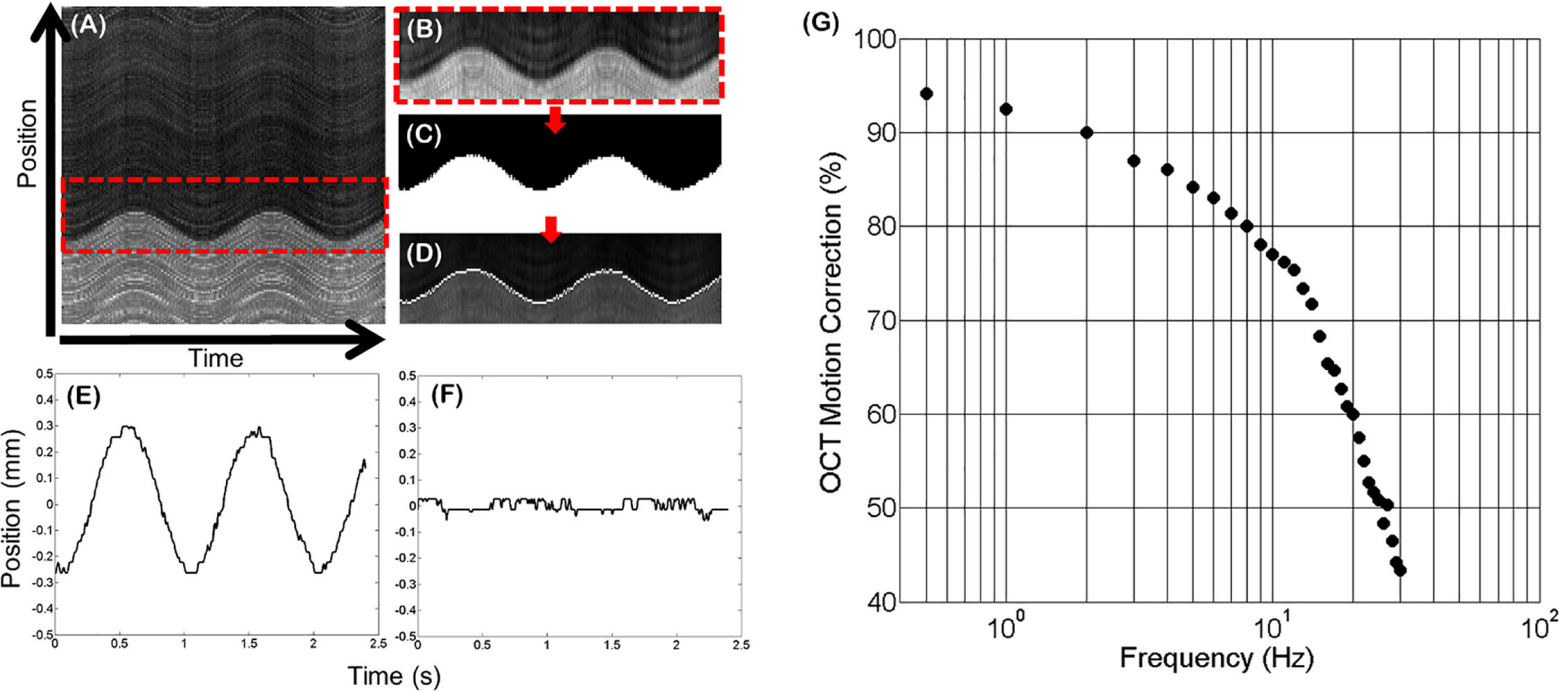
Characterization of motion correction bandwidth. (A) 2D M-scan acquired during simulated pupil phantom motion without pupil tracking correction. The red box shows the segmented pupil edge. The segmented portion was then smoothed with a Gaussian filter (B) and intensity thresholded (C). Edge detection (D) yielded the pupil motion trace. Motion traces without (E) and with (F) pupil tracking are shown for 1 Hz simulated sinusoidal motion. (G) Motion correction percentage as a function of simulated motion frequency.

To characterize the frequency response of the pupil tracking system integrated into the ASOCT system and thus estimate the OCT motion correction bandwidth, the FSM was used to simulate pupil phantom motion with sinusoidal waveforms at varying frequencies (.5–30 Hz) and constant amplitude (corresponding to 1 mm simulated lateral motion of the phantom). OCT 2D M-scans composed of 240 A-lines/B-scans (with a length of 8 mm) and 1000 B-scans were acquired at the same lateral position on the pupil phantom. The B-scan acquisition rate was 416.7 Hz, yielding a 2D M-scan acquisition time of 2.4 seconds. Each 2D M-scan was summed in the axial dimension and the pupil edge in the resulting summed voxel projection (SVP) was segmented, smoothed using a 3x3 Gaussian filter, and thresholded to produce a binary image. Sobel edge detection was used to segment the edges of the binary and generate an OCT motion trace of the simulated motion imparted on the pupil phantom (Fig 5). Motion traces generated from the OCT 2D M-scans with and without pupil tracking and real-time correction of the ASOCT scanning waveforms were acquired at each input frequency. The ratio of the amplitudes of the uncorrected and corrected motion traces served as a metric for percentage of motion corrected at each frequency, and thus generated the pupil tracking ASOCT system transfer function. The adjustable gain parameter *β* was optimized by minimizing the residual motion present in the corrected motion trace at 0.5 Hz of sinusoidal simulated motion. The OCT motion correction percentage as a function of frequency was then calculated and the resulting frequency response is shown in Fig 5. The -3 dB amplitude roll-off of the transfer function was 22 Hz.

### Pupil tracking ASOCT imaging

**Fig 6:**
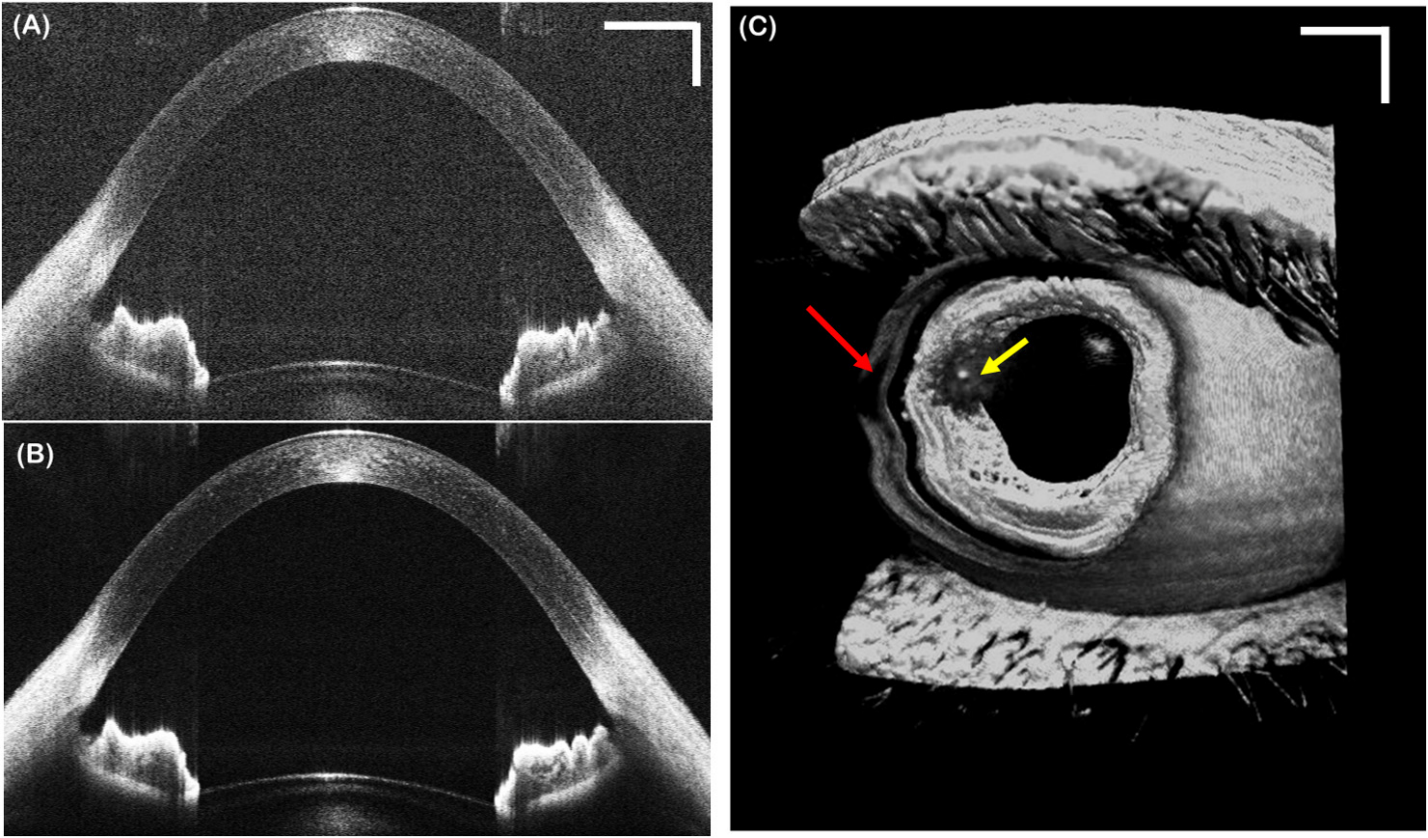
Representative anterior segment OCT motion images acquired from a healthy volunteer without tracking. (A) Single frame B-scans composed of 1000 A-lines. (B) Registered and averaged (10x) B-scan acquired in repetitive B-scan mode at a frame rate of 100 Hz. (C) Volumetric image composed of 1000 A-lines and 200 B-scans, corresponding to a volume frame rate of 0.5 Hz. Red arrow denotes significant motion artifacts due to patient motion. Scale bars are 1 mm. Yellow arrow denotes an artifact from specular reflection.

To test the ASOCT system, images of healthy subjects were first acquired without tracking. Fig 6 shows a representative single frame B-scan (Fig 6(A)), an averaged (10x) and registered B-scan (Fig 6(B)) composed of repetitive B-scans acquired at the same lateral position, and a volumetric image (Fig 6(C)). The B-scans parameters were 1000 A-scans/B-scan, corresponding to 100 Hz frame rate. The volume consisted of 1000 A-scans/B-scan and 200 B-scans per volume, corresponding to a volume rate of 0.5 Hz. The ASOCT system achieved sufficient imaging depth and sensitivity to visualize the entire anterior chamber (from anterior cornea to anterior lens). The red arrow on the volume denotes motion artifacts caused by patient motion during the scan acquisition, which cannot be corrected using B-scan registration.

**Fig 7:**
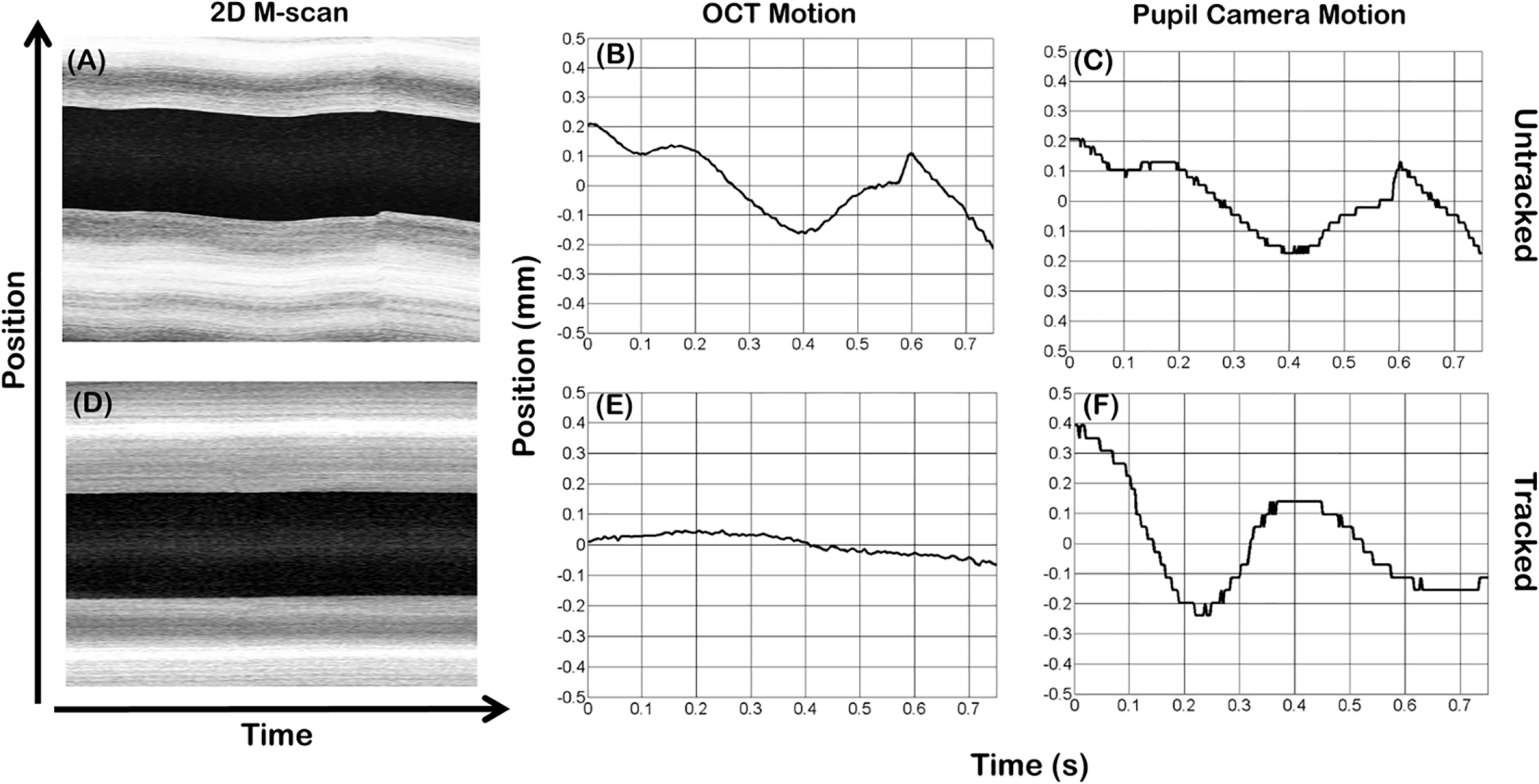
*In vivo* 2D M-scans acquired from a healthy volunteer with and without tracking. (A-C) Untracked SVP and motion traces extracted from the 2D M-scan and pupil camera. (D-F) Tracked SVP and motion traces extracted from the 2D M-scan and pupil camera.

To demonstrate the utility of *in vivo* pupil tracking ASOCT, 2D M-scans were acquired from healthy volunteers with and without tracking (Fig 7). The 2D M-scans were composed of 600 A-scans/B-scans and 125 B-scans acquired at the same spatial location, resulting in a B-scan frame rate of 166.67 Hz covering a 10 mm field of view. SVPs of the 2D M-scans were generated and displayed to show lateral pupil motion, as shown by the “dark band” corresponding to the pupil. The OCT lateral motion along the fast scan axis between successive B-scans was extracted from the 2D M-scans by cross-correlating sequential B-scans. The subject’s pupil motion was also recorded using the pupil camera. Without tracking, the OCT motion estimated using B-scan cross-correlation correlated well with the detected pupil motion. With tracking activated, the standard deviation of the motion trace throughout the 0.75 s acquisition time was reduced from 116 μm (without tracking) to 33.8 μm (with tracking).

**Fig 8:**
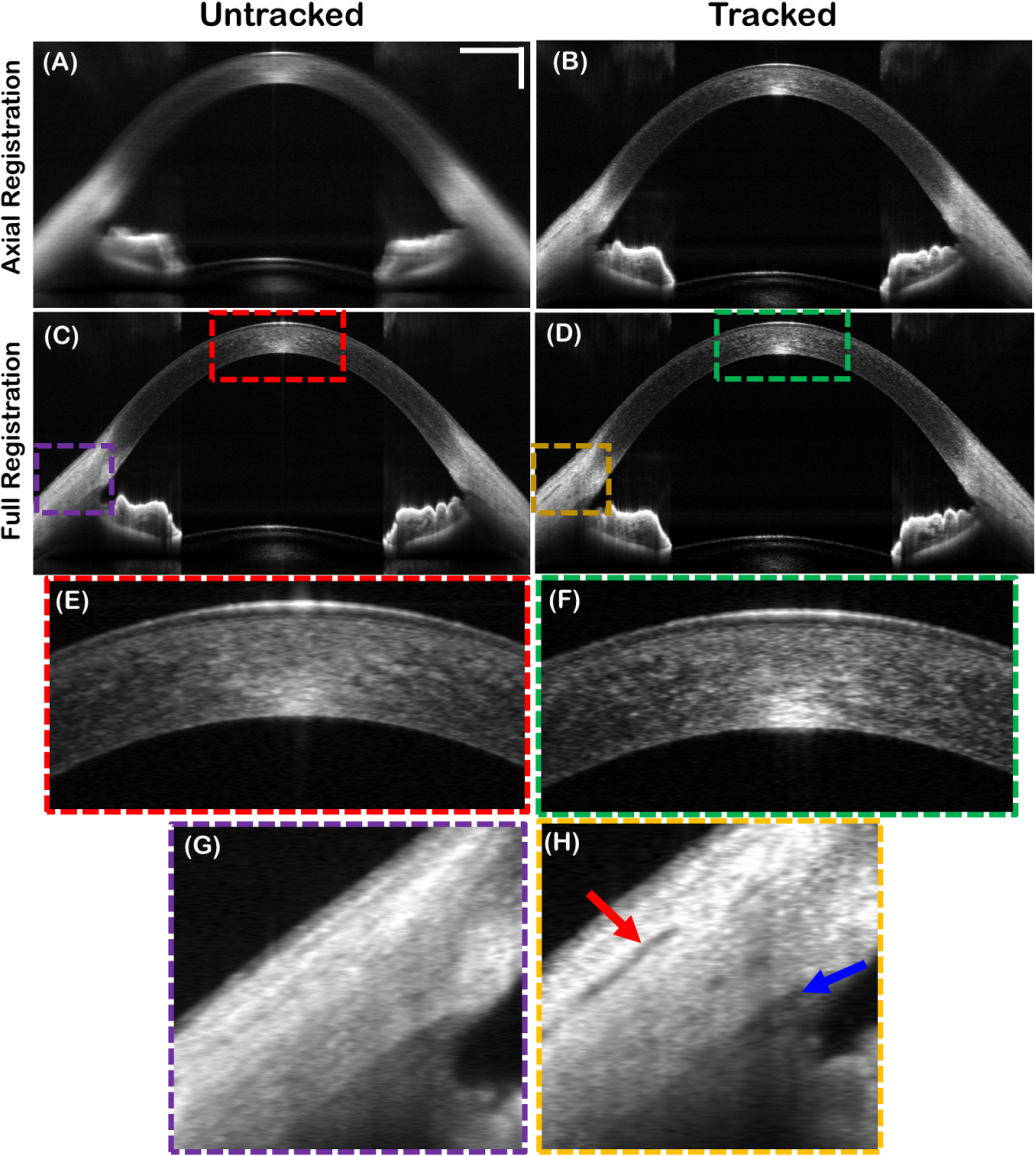
Averaged B-scans generated by summing 125 B-scans acquired at the same location in 0.75 seconds with and without tracking. (A-B) Averaged, untracked and tracked B-scans after axial registration only. (C-D) Averaged, untracked and tracked B-scans after full (lateral+axial) registration. (E-F) Digitally zoomed images of corneal stroma for untracked and tracked fully registered B-scans. (G-H) Digitally zoomed images of the anterior chamber angle. Scleral striations (red arrow) and Schlemm’s canal (blue arrow) were better resolved with tracking. Scale bars are 1 mm.

The 125 B-scans corresponding to the 2D M-scans above were axially registered, summed, and displayed in Fig 8A and 8B. Pupil tracking mitigated motion artifacts and preserved lateral resolution. Fully (axial + lateral) registered B-scans for the untracked and tracked scenarios are also shown (Fig 8C and 8D). Even after full registration, tracking resulted in higher quality averaged images due to tracked patient motion orthogonal to the B-scan axis. That is, B-scan registration only compensated for patient motion in the fast scan and axial dimensions, since the cross-correlations for registration were calculated from the acquired B-scans. In contrast, pupil tracking compensated for 2D lateral motion in real-time.

**Fig 9:**
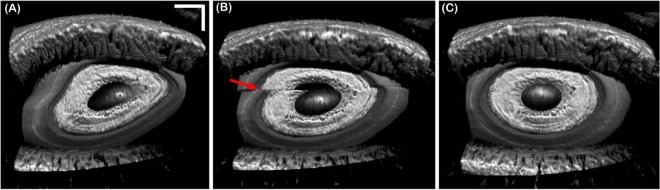
Anterior segment volumetric time series acquired with volumes composed of 500 A-lines/B-scans and 200 B-scans/volume with and without tracking. (A) Volume corrupted by prominent motion artifacts before activating tracking. (B) Volume during which tracking was activated; image artifact caused by repositioning of the scanners is denoted by the red arrow. (C) Volume acquired with tracking activated. Scale bars are 1 mm.

Motion compensation during volumetric OCT acquisition was tested by acquiring a series of 10 consecutive volumes composed of 500 A-lines/B-scan and 200 B-scans/volume from a healthy subject during fixation, corresponding to a total imaging time of 10 seconds. Tracking was activated during the acquisition. Fig 9 shows 3 consecutively acquired volumetric images. The first volume (Fig 9(A)) was acquired before tracking was turned on and therefore was corrupted by motion. Tracking was turned on during the acquisition of the next volume (Fig 9(B)) and resulted in an artifact due repositioning of the scanners (red arrow). Fig 9(C) shows a volume free of motion artifacts acquired with tracking engaged.

## Discussion

Previous studies reported the power spectrum of fixational human eye motion to follow a 1/*f* [32,33,29] or 1/*f*^2^ [34] distribution in which the majority of motion amplitude was restricted to below 10 Hz. Additional potential sources of motion such as operator/subject misalignment and the subject’s inability to fixate are slow compared to fixational motion. The current iteration of our pupil tracking system corrects eye motion at up to 10 Hz and is thus sufficient to compensate for the majority of motion that could be present during ASOCT imaging. The correction bandwidth of the system can be improved by using GPU-accelerated pupil segmentation software to minimize system latency, which has a significant impact on the tracking performance as discussed in detail below.

The discrepancy between the frequency response function measured using the step response (Fig 4(C)) of the tracking algorithm and the OCT motion correction frequency response (Fig 5(G)) can be attributed to the measured latency of the control loop. To understand the impact of latency on the tracking system, subject motion at the OCT imaging plane and the corresponding correction signal generated by the tracking algorithm were modeled using two sinusoidal functions with amplitude *A*, frequency *f*, and a relative phase offset corresponding to a latency of Δt:

x(t)=Asin⁡(2πft) (2)

y(t)=−Asin⁡(2πf(t+Δt)) (3)

where *x*(*t*) is the subject motion and *y*(*t*) is the tracking system output. The amplitude of motion that is not corrected by the tracking system was modeled by summing *x*(*t*) and *y*(*t*), which yielded:

x(t)+y(t)=2Asin(πfΔt)cos⁡(πf(2t+Δt)). (4)

**Fig 10:**
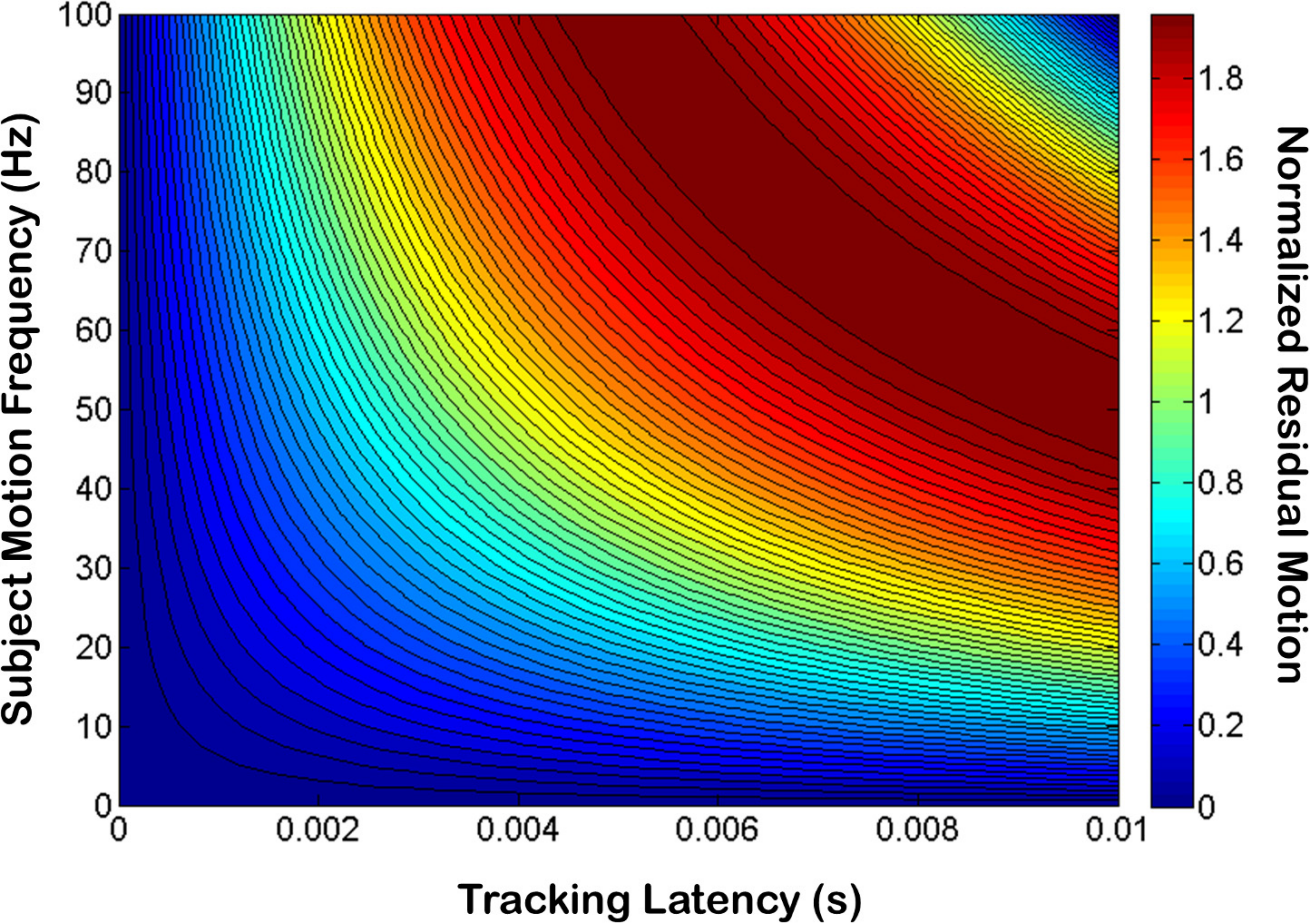
Uncorrected residual motion (normalized by subject motion) as a function of latency and motion frequency. For a latency of 4 ms, our model predicted a -3dB roll-off at 20 Hz for the OCT motion correction bandwidth, which was close to our measured value of 22 Hz.

The amplitude modulation in Eq 4 represents the amplitude of residual motion present *after* tracking was applied and could be used to model the motion rejection transfer function of the tracking system as a function of both subject motion frequency and latency. The residual amplitude 2*A*sin(*πf*Δ*t*) normalized by the input subject motion amplitude *A* was plotted for Δ*t* = 0–10 ms and *f* = 0–100 Hz (Fig 10). Our model predicted that for a latency of 4 ms, the residual motion amplitude should be 50% of the subject motion amplitude at 20 Hz, which is close to our measured value of 22 Hz (Fig 5(G)). From this graph we also see that due to latency, tracking could result in amplification instead of mitigation of subject motion (i.e, residual motion > 1) for some frequencies. In our system which exhibited a latency of 4 ms, motion amplitude at frequencies above 50 Hz could be amplified; however, we may not have noticed the effects of this amplification because the magnitude of motion at these frequencies might have been negligible.

## Conclusions

We have demonstrated a pupil tracking system for real time motion compensated ASOCT. Pupil oculography hardware coaxial with the swept-source OCT enabled fast detection and tracking of the pupil centroid. The pupil tracking ASOCT system with a field of view of 15 x 15 mm achieved diffraction-limited imaging over a lateral tracking range of +/- 2.5 mm and was able to correct eye motion at up to 22 Hz. Pupil tracking ASOCT offers a novel real-time motion compensation approach that may facilitate accurate and reproducible anterior segment imaging.
